# Understanding the Multidimensional and Dynamic Nature of Facial Expressions Based on Indicators for Appraisal Components as Basis for Measuring Drivers' Fear

**DOI:** 10.3389/fpsyg.2021.622433

**Published:** 2021-02-18

**Authors:** Meng Zhang, Klas Ihme, Uwe Drewitz, Meike Jipp

**Affiliations:** Institute of Transportation Systems, German Aerospace Center/Deutsches Zentrum für Luft- und Raumfahrt (DLR), Braunschweig, Germany

**Keywords:** fear, facial expression, action units, in-vehicle, component process model

## Abstract

Facial expressions are one of the commonly used implicit measurements for the in-vehicle affective computing. However, the time courses and the underlying mechanism of facial expressions so far have been barely focused on. According to the Component Process Model of emotions, facial expressions are the result of an individual's appraisals, which are supposed to happen in sequence. Therefore, a multidimensional and dynamic analysis of drivers' fear by using facial expression data could profit from a consideration of these appraisals. A driving simulator experiment with 37 participants was conducted, in which fear and relaxation were induced. It was found that the facial expression indicators of high novelty and low power appraisals were significantly activated after a fear event (high novelty: *Z* = 2.80, *p* < 0.01, *r*_*contrast*_ = 0.46; low power: *Z* = 2.43, *p* < 0.05, *r*_*contrast*_ = 0.50). Furthermore, after the fear event, the activation of high novelty occurred earlier than low power. These results suggest that multidimensional analysis of facial expression is suitable as an approach for the in-vehicle measurement of the drivers' emotions. Furthermore, a dynamic analysis of drivers' facial expressions considering of effects of appraisal components can add valuable information for the in-vehicle assessment of emotions.

## Introduction

Over the past decade, affective computing came into the focus of research for driver monitoring systems, because some emotions are supposed to impact drivers' cognitive capabilities necessary for driving and risk perception (Jeon et al., 2011). Therefore, detecting and mitigating driver emotions by using affective computing in an emotion-aware system may ensure driving safety (Ihme et al., 2019). One idea of such a system is to interpret the user's emotional state and provide assistance to support users to reduce the negative consequences of certain emotional states (Klein et al., 2002; Tews et al., 2011; Jeon, 2015; Löcken et al., 2017; Ihme et al., 2018). Furthermore, in the context of high-level automated driving functions, an automated assessment of emotions could allow adapting driving styles or warnings to the drivers' current emotional state to maximize drivers' comfort and optimize the driving experience (Techer et al., 2019). For instance, fear, which refers to the emotional responses evoked by processing threatening stimuli (Schmidt-Daffy et al., 2013), can be regarded as an indicator of experienced risk (Fuller, 1984). Hence, the recognition of fear could help automated driving functions to adapt their speed to reduce the feeling of risk and regain trust on it. However, theoretically, emotions usually have been regarded as a static state rather than a dynamic process (Scherer, 1984, 2019). Accordingly, emotions' time courses and the underlying mechanism have been barely focused in practical applications, especially in the driving context, in which a dynamic interpretation of drivers' spontaneous emotion is required. Interestingly, a recent study confirmed that the recognition of emotions from dynamic facial expressions was more accurate than from static ones (Namba et al., 2018), which suggests considering the multidimensional and dynamic nature of emotions for affective computing may increase the possibility for a practical implementation of emotion-aware systems. Therefore, investigating the multidimensional and dynamic nature of drivers' emotion would contribute to the development of reliable in-vehicle emotion measurement.

The Component Process Model (CPM) provides a comprehensive theoretical framework for the multidimensional and dynamic interpretation of emotions (Scherer, 1984, 2009a; Scherer et al., 2019). According to the CPM, a given situation would be appraised with multidimensional criteria (the appraisal components), which would follow a fixed order. Furthermore, the result of the individual's appraisals would impact the different components autonomic physiology, action tendencies, motor expressions and subjective feeling (Scherer, 2009b). There are four main appraisal components, which were supposed to happen in sequence: 1, novelty, which means, how sudden or unfamiliar the individual perception of the given situation is; 2, pleasantness, which represents positive or negative feelings about the given situation; 3, goal significance, which represents the impact of the situation on an individual goal; 4, coping potential/power, which represents whether the situation is controllable. Particularly, the appraisal components of pleasantness and power are assumed to be the determinants of valence and power suggested by dimensional emotion theorists (Scherer et al., 2019). The appraisal component of novelty, on the other hand, is suggested to be an additional dimension to the dimensional emotion space (Scherer, 2009b). In the assumption of the CPM, the results of these appraisal components would specifically impact the autonomic nervous system (e.g., changing in heart rate) and somatic nervous system (e.g., changing in facial expressions or voice) (Scherer et al., 2019). Thus, multidimension and dynamics in facial expressions and autonomic nervous system activity can be used as indicators for the presence of certain appraisal processes rendering multidimensional and dynamic interpretation of emotion possible.

Implicit measurements are required for the in-vehicle affective computing. In previous studies, drivers' emotions have been assessed by using voice (Abdic et al., 2016) or facial temperature (Zhang et al., 2019). Besides, facial expressions were one of the commonly used implicit measurements of drivers' emotion (Malta et al., 2011; Abdic et al., 2016; Ihme et al., 2018). Specifically, camera-based approaches of facial expression analysis appear suitable for in-vehicle emotion collection, because these are contactless and unobtrusive. However, up to now, approaches for in-vehicle assessment of emotions based on facial expressions neglect the time courses of and the mechanisms underlying the facial expressions. According to the CPM, it is assumed that the occurrence of a facial expression is a sequential-cumulative process, which is triggered by appraisal components in sequence (Scherer et al., 2018). For instance, fear can be interpreted as an emotion with high novelty and low power (Scherer et al., 2018). Thus, a fearful facial expression may firstly consist of a raised eyebrow representing “unpredictable” and then a dropped jaw representing “out of control.” The Facial Action Coding System (FACS, Ekman and Friesen, 1978; Ekman et al., 2002) can be used to describe facial expressions systematically based on activity in atomic units of facial action, the action units (AUs). Interestingly, a recent paper by Scherer et al. (2018) integrates empirical evidence to determine the relationship between activation in certain AUs and appraisal components based on the FACS (see Table 1). To add, in a facial electromyography (EMG) study by Gentsch et al. (2015), corrugator and frontalis regions were revealed to indicate goal significance, while activity in the cheek was supposed to be influenced by coping potential/power. Furthermore, the study suggested that appraisal components drive facial expressions in a fixed sequence and that the effects of power appraisal follow goal significance. Still, besides the work by Gentsch et al. (2015), the empirical evidence for this approach is scarce, so that it needs to be verified especially for the assessment of emotions in applied settings. Therefore, the aim of this study was to investigate whether multidimensional analysis of facial expression is a suitable approach for the in-vehicle measurement of the drivers' emotions. Furthermore, the possibility of dynamic analysis of drivers' facial expressions considering effects of appraisal components is investigated. In this study, we chose fear as the target emotion, because recognition of fear could help to adapt the driving style of automated vehicles to reduce the subjective feeling of risk. In order to present the distinct time difference between appraisals, we focused on the first and last appraisal components results: high novelty and low power to reveal the dynamic process in facial expressions of fear. For this, we induced fear as experimental condition and relaxation as control condition in a realistic driving simulation and extracted participants' facial expressions from camera recordings. Based on the aforementioned considerations, we assumed that the activation in specific AUs (1, 2, 4, 5, and 7) indicates high novelty and low power. We also assumed that activation in specific AUs (15, 20 25, and 26) indicating low power follow the activation in AU indicating high novelty.

**Table 1:** On basis of CPM prediction of appraisal and action units (AU) for fear (adapted table from Scherer et al., 2018).

**Cumulative sequence of appraisal**	**Appraisal components**	**Appraisal predicted results**	**AUs predicted**
1	Novelty	high	1, 2, 4, 5, 7, 26, 38
2	Pleasantness	open	
3	Goal significance	high	4, 7, 23, 17
4	Power	low	1, 2, 5, 15, 20, 25, 26

*AU: 1 inner brow raiser, 2 outer brow raiser, 4 brow lowerer, 5 upper lid raiser, 7 lid tightener, 15 lip corner depressor, 17 chin raiser, 20 lip stretcher, 25 lips part, 26 jaw drop, 38 nostril dilator*.

## Materials and Methods

### Design

The two target emotional states (fear and relaxation) were induced during two automated driving scenarios in a within-participants design. We assessed participants' facial muscle activity from camera recordings based on the FACS.

### Participants

In total, 50 volunteers took part in this driving simulator study. All of them had a Western European cultural background, lived in Northern Germany and had German as first language. Thirty-seven of these [thirteen females, age range from 18 to 62 years, mean (*M*) = 31 years, standard deviation (*SD*) = 11 years] completed the relevant emotion-induction experimental sessions and their faces were validly recorded on camera, so that they could be included into the data analyses. Thirteen participants were excluded because of incomplete self-report questionnaires (three) and due to technical problems with the face detection from the video signals (ten).

Before the start of the study, the participants were informed about the video recording, potential risks of driving in simulators (e.g., the experience of simulator sickness) according to the simulator safety concept and the rough duration of the experiment. The participants were informed that they could take a break or abort their participation at any time. All participants provided written informed consent to take part in the study and the video recording. As reimbursement for their time, the participants received 10 € (12 $) per commenced hour for their participation. After finishing, the participants were informed about the true goal of the experiment (evoking certain emotions) and the necessity to conceal this goal with a cover story (see below).

### Set-Up

The study took place in the DLR's Virtual Reality (VR) laboratory consisting of a realistic 360° projection and steering wheel as well as gas and brake pedals. Video data of the participants' faces were recorded from the front with a network camera (Abus, Wetter, Germany) with a frame rate of 15 frames per second and a resolution of 1,280 × 720 pixels. In order to reduce the influence of changing light and ensure constant lighting, an LED band was mounted above participants' head. The driving simulation was realized using the Virtual Test Drive software from Vires (Vires Simulationstechnologie, Bad Aibling, Germany).

### Procedure and Scenario

**Figure 1:**
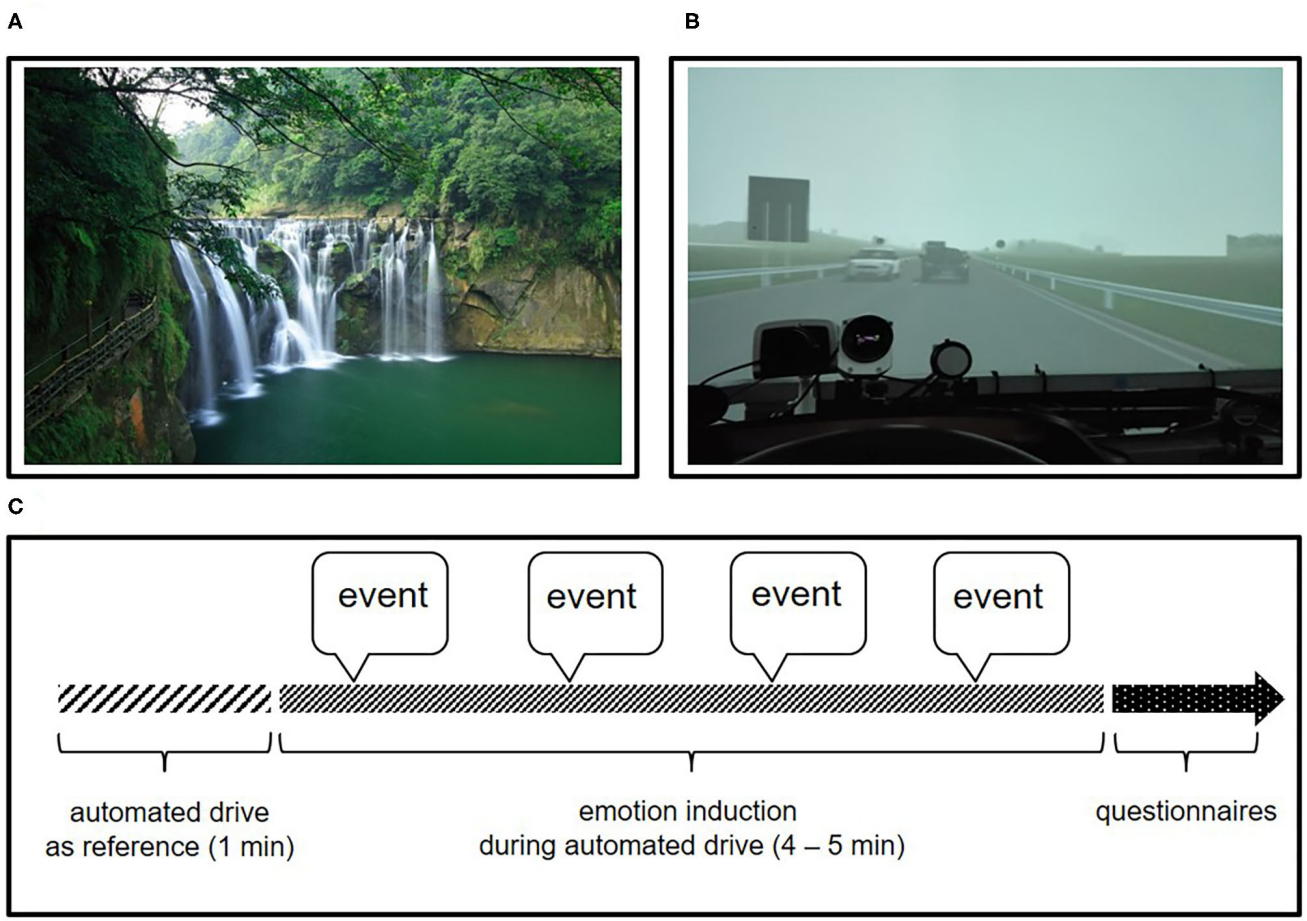
**(A)** Example for a relaxing image from the baseline scenario (Chiu, 2006); **(B)** View of participants while a vehicle swerving abruptly from the opposite lane during fear scenario; **(C)** Sketch of the procedure of each trial.

With the instructions for the experiment, the participants were presented with a cover story which was supposed to obscure the true background of the study (induction and measuring of emotion). According to the cover story, the aim of the study was to investigate the influence of secondary tasks during an automated drive on the driving performance during a subsequent manual drive. Six emotional states (fear, frustration, joy, sadness, surprise and uncertainty) and relaxation were to be induced in the experiment and the respective tasks to trigger the emotions were included into the cover story. During the experiment, the participant sat alone in the cockpit of the vehicle mock-up and experienced all emotion induction phases in sequence (during the breaks between the drives, the participants had contact to the experimenter). The presentation order of the emotion induction phases was randomized across the participants to reduce the impact of potential ordering effect. For the induction, an automated driving scenario was used, in which the participants where driven by the car in automated driving mode at a given speed along a given route. The drive always started 5 s after the corresponding scenario was activated, with the drive going smoothly for the first minute without any emotional event happening. This period was used to collect a reference for the emotion induction afterwards. Then, the emotional events took place at the given time in the rest of drive (see Figure 1C). Here, we focused on fear and relaxation, which were, respectively, regarded as the experimental condition (Fear) and baseline condition (BL).

The fear induction took place on a route of 6 km (3.7 mi) length consisting of a highway section with three lanes and a country road section with one lane per direction. At 1:47 and 3:54 min after the beginning of the scenario, the automated driving vehicle was involved in an accident, which was caused by a vehicle swerving abruptly from the opposite lane (see Figure 1B). Both events were associated with loud noise (collision and loud braking). In addition, in order to distract the participants and to enhance the experienced fear, a text message in the form of an SMS was presented 5 s before the accident on the right in the field of vision during the drive. The drive had a total duration of ~285 s. In this paper, the first event of the experimental condition was considered for further analysis, because it was expected to induce more intensive emotion than the subsequent events; furthermore, the onset time of the first event was comparable between control and experimental condition.

The scenario of relaxation took place on a 4 km (2.5 mi) country road with one lane per direction. Relaxation was supposed to be induced using the large-scale presentation of nature photographs as events. Four large-scale images, each with a presentation time of 50 s, were shown (see Figure 1A). The presentation was accompanied by relaxing music. The journey had a total duration of ~265 s. Again, the first event, which was presented after 1 min of driving, was considered for further analysis.

### Self-Report Questionnaires

After each driving scenario, the participants were asked to complete self-report questionnaires to assess their emotional experience during the drives. For this, we used the Positive and Negative Affect Schedule (PANAS) and an adapted version of Self-Assessment Manikin (SAM, Bradley and Lang, 1994).

The PANAS [Original: Watson et al., 1988; German version: Krohne et al., 1996] is composed of 20 adjectives describing ten positive and ten negative emotions, on a Likert scale (1 - very slightly, 2 - a little, 3 - moderately, 4 - quite a bit, 5 - extremely). We focused our analysis on the “scared” and “relaxed,” which were semantically closest to our target emotions.

The SAM uses pictures to represent emotional responses on the three dimensions valence (pleasure-displeasure), arousal (calm-activity) and dominance/power (control-out of control). A question of experienced novelty was additionally added as the fourth dimension. Each dimension was represented by a Likert scale from one to nine (1 - very slightly to 9 - extremely).

### Action Units

For extracting the frame-to-frame activity of the facial AUs, we used the Attention Tool FACET Module (FACET, iMotions), which is a face and AU detection software based on the FACS. This software can track and quantify changes in AUs frame by frame and was validated in studies comparing with human coders (Krumhuber et al., 2019) and comparing with facial Electromyography (EMG) recording (Kulke et al., 2020). ~300,000 frames (37 participants * (285 + 265 s) * 15 Hz) were encoded with FACET, whereby AU 1, 2, 4, 5, 6, 7, 9, 10, 12, 14, 15, 17, 18, 20, 23, 24, 25, 26, and 28 were the variables. Each AU was assigned a numerical value, which is originally called evidence in the FACET software. For better understanding, we use the term AU index in the remainder of this work. The AU index is a raw data value that relates to the likelihood of an AU occurring. In order to reduce the difference between the participants, all output of encoding was scaled within every drive and adjusted, whereby the average AU index of the AUs in the first minute of the respective drive was subtracted from the AU index of the AUs in the experimental or control condition.

In order to quantify the changing in different components, we used the linear average value of certain relevant AUs as a compound measure to indicate components. We assumed that using of compound of AUs could increase the signal-to-noise ratio of the appraisal components. In a previous EMG study, facial muscle activity in the frontalis region was revealed to be related with the appraisal component of novelty (Sequeira et al., 2009). According to this assumption, the prediction of the CPM (Scherer et al., 2018, Table 1) and avoiding overlap between components, the compound (linear average) of upper facial AUs 1, 2, 4, 5, and 7 was used to indicate the appraisal component of high novelty and the compound of the lower facial AUs 15, 20, 25, and 26 for the appraisal component of low power (see Table 2 for a semantic description of the AUs). AU 38 was not used because it was not covered by the software package used for facial AU analysis.

**Table 2:** Description of action units, which were used in analysis.

**AU**	**Description**
1	Inner brow raiser
2	Outer brow raiser
4	Brow lowerer
5	Upper lid raiser
7	Lid tightener
15	Lip corner depressor
20	Lip stretcher
25	Lips part
26	Jaw drop

In order to reveal the temporal dynamics on both components, the AU compounds were segmented from event onset to 5 s after event onset (BL: picture presentation, Fear: Swerving vehicle occurrence). On one hand, the mean value of the AU compounds in 5 s were calculated and compared between Fear and BL. On the other hand, the means were also aggregated in subsequent windows of 100 millisecond length in order to reveal the changing over time.

### Statistical Analyses

According to the results of Shapiro-Wilk normality tests, neither subjective rating's data nor the mean value of AU compounds were normally distributed (dimension of novelty: W = 0.94, *p* < 0.01; dimension of valence: *W* = 0.90, *p* < 0.001; dimension of arousal: *W* = 0.87, *p* < 0.001; dimension of power: *W* = 0.92, *p* < 0.001; PANAS- “scared”: *W* = 0.74, *p* < 0.001; PANAS- “relax”: *W* = 0.9, *p* < 0.001; AU Compound of high novelty: *W* = 0.96, *p* < 0.05; AU Compound of low power: *W* = 0.90, *p* < 0.001). Therefore, a Wilcoxon test for dependent samples was implemented for the comparison between Fear and BL and the results were presented as Z-score. The condition with two levels Fear and BL was the only factor. The significance level of α = 0.05 was used for the overall test. For determining the effect size, the computational parameter *r*_*contrast*_ recommended by Rosenthal et al. (1994) was used. Hereby, the effect size is low if *r*_*contrast*_ <0.1, medium if *r*_*contrast*_ <0.3 and large if *r*_*contrast*_ >0.5.

In order to identify time points at which the AU compound between Fear and BL begin to diverge, the data in the time interval of 5 s were analyzed pointwise by F-tests, which is believed to provide relevant rather than trivial differences between two functional linear models (Shen and Faraway, 2004). Using the “ERP” package (Causeur et al., 2014) with Benjamini-Hochberg (BH) procedure (Benjamini and Hochberg, 1995) in the R programming language, it was ensured that the false discovery rate (FDR) was controlled at a preset level α.

## Results

### Manipulation Check

**Figure 2:**
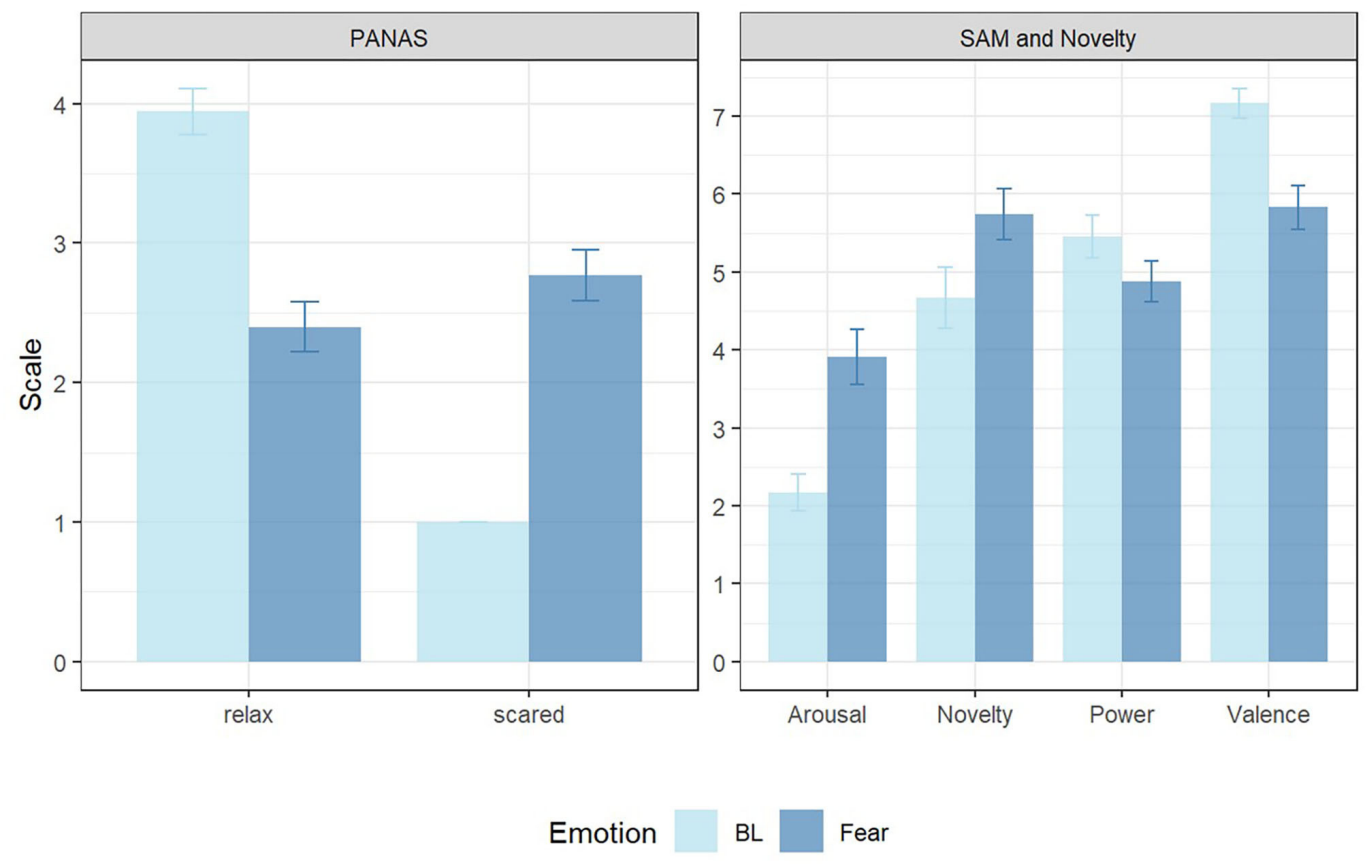
Mean and standard error of ratings in PANAS “relax” and “scared” (left) as well as in SAM scales and the added dimension of novelty (right) in Baseline (BL, light blue) and Fear (dark blue).

The participants' rating on the PANAS item “relax” was significantly higher in the BL scenarios than in the Fear scenarios according to a Wilcoxon test for dependent samples (*Z* = −4.27, *p* < 0.001, *r*_*contrast*_ = 0.7). On the contrary, the rating on the PANAS item “scared” was significantly higher in the fear scenarios comparing the BL scenarios (*Z* = −4.83, *p* < 0.001, *r*_*contrast*_ = 0.79) (see Figure 2). Furthermore, the ratings on PANAS item “surprised,” “alert,” “active,” “insecure,” “attentive,” “upset,” “afraid,” “interested,” “nervous,” “frustrated,” “jittery,” “distressed,” “determined” “angry” and “ashamed” were also significantly higher in Fear (see Table 3). Significant differences between Fear and BL scenarios were also found in the SAM dimensions arousal (*Z* = 3.51, *p* < 0.001, *r*_*contrast*_ = 0.58) and valence (*Z* = −3.43, *p* < 0.001, *r*_*contrast*_ = 0.56). No significant difference was found for the rating on SAM's power dimension. However, a trend for a difference was revealed (*Z* = −1.94, *p* = 0.053, *r*_*contrast*_ = 0.32). Additionally, the participants' rating on the dimension novelty was significantly higher in the Fear scenarios according to a Wilcoxon test for dependent samples (*Z* = 2.45, *p* < 0.05, *r*_*contrast*_ = 0.4) (see Figure 2).

**Table 3:** The rating on PANAS (descending ordered by the magnitude of difference between Fear and BL).

**Items**	***M* (Fear)**	***M* (BL)**	***M* (Fear - BL)**	**Z**
Scared	2.77	1.00	1.77	4.83^***^
Surprised	2.94	1.36	1.58	4.76^***^
Alert	3.77	2.64	1.13	4.58^***^
Active	2.97	1.92	1.05	3.94^***^
Insecure	2.26	1.22	1.04	3.88^***^
Attentive	3.83	2.97	0.86	3.89^***^
Upset	1.77	1.00	0.77	3.63^***^
Afraid	1.77	1.03	0.74	3.45^***^
Interested	3.09	2.42	0.67	3.28^**^
Nervous	1.91	1.25	0.66	3.55^***^
Frustrated	1.69	1.06	0.63	3.85^***^
Jittery	1.63	1.08	0.55	2.76^**^
Distressed	1.60	1.14	0.46	2.14^*^
Determined	2.74	2.31	0.43	2.64^**^
Angry	1.43	1.00	0.43	2.11^*^
Ashamed	1.29	1.00	0.29	2.13^*^
Sad	1.17	1.14	0.03	0.22
Proud	1.31	1.47	−0.16	−1.56
Enthusiastic	1.91	2.14	−0.23	−1.17
Inspired	1.71	1.97	−0.26	−1.01
Excited	1.60	2.08	−0.48	−2.06^*^
Relax	2.40	3.94	−1.54	−4.27^***^

* p < 0.05;

** p < 0.01;

*** *p < 0.001*.

### Action Units Compounds

**Figure 3:**
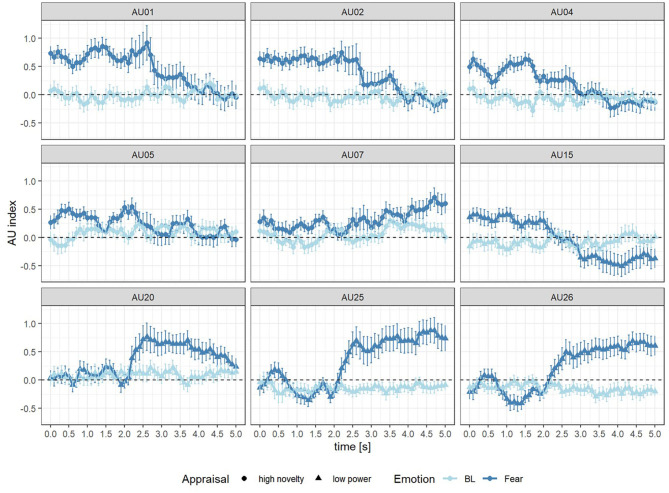
AU index of Fear (dark blue) and Baseline (BL, light blue) in 0–5 s after the onset of event for AU 1, 2, 4, 5, and 7 (high novelty) as well as AU 15, 20, 25, and 26 (low power).

Figure 3 shows the changing of relevant AUs in subsequent windows of 100 millisecond length for 5 s after event onset. Generally, there was difference between the changing in Bl and Fear on several AUs: AU 1, 2, 4, 5 as well as 15 were activated more in Fear than BL before 2.5 s, while after 2.5 s AU 20, 25, and 26 were activated more in Fear than BL.

The linear average of the changing of the compounds for high novelty (AU 1, 2, 4, 5, and 7) and low power (AU 15, 20, 25, and 26) was calculated for 5 s after event onset. The Wilcoxon test for dependent samples indicated that the changing of novelty was significantly higher in Fear scenario (*Z* = 2.80, *p* < 0.01, *r*_*contrast*_ = 0.46). Significant differences between Fear and BL scenarios were also found in compound low power (*Z* = 2.43, *p* < 0.05, *r*_*contrast*_ = 0.50).

**Figure 4:**
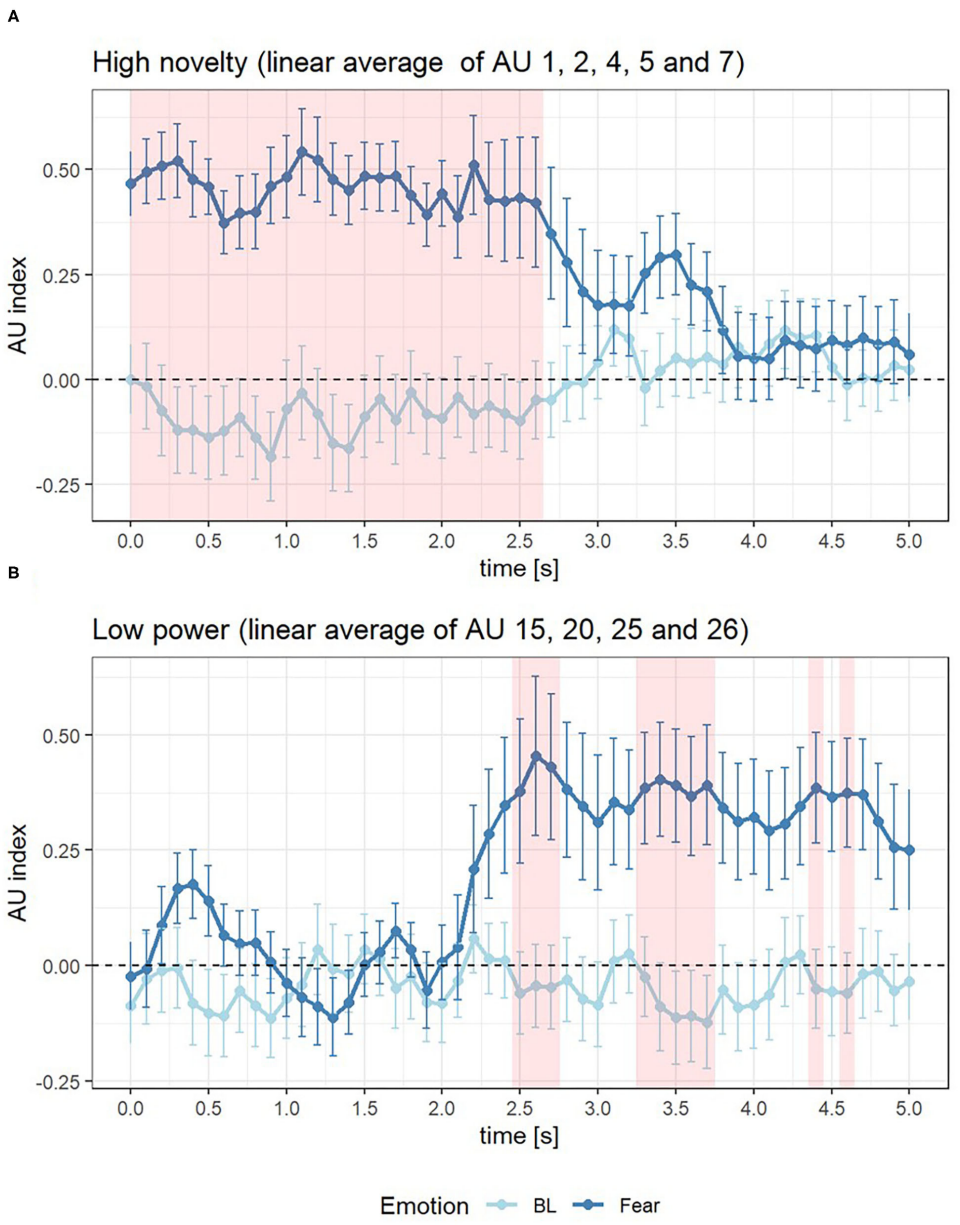
AU index of Fear (dark blue) and Baseline (BL, light blue) in 0–5 s after the onset of event for component of high novelty **(A)** and low power **(B)**, where the red area represents the time interval when the AU index difference between Fear and BL >0.

For the dynamics of the compounds high novelty and low power, the following results were obtained: The AU compound of high novelty was continuously significantly activated from 0 to 2.6 s after the onset of Fear compared to BL events (see Figure 4A and Table 4). The activation of the AU compound of low power started at 2.5 s, at which the difference between Fear and BL was significant (*M* = 0.44, *F* = 2.99, *p* < 0.05). Activation of the AU compound of low power in Fear could be discontinuously found between 2.5 and 4.6 s after event onset (see Figure 4B and Table 4).

**Table 4:** Mean AU index difference of Fear and BL (*M*) and results of F-tests (*F*) for each time point of high novelty (AU 1, 2, 4, 5, and 7) and low power (AU 15, 20, 25, and 26).

**High novelty (Fear - BL)**						**Low power (Fear - BL)**					
**Time**	***M***	***F***	**Time**	***M***	***F***	**Time**	***M***	***F***	**Time**	***M***	***F***
0.0	0.47	3.93^**^	2.6	0.47	2.52^*^	0.0	0.06	0.56	2.6	0.50	3.30^*^
0.1	0.51	3.97^**^	2.7	0.40	2.10	0.1	0.02	0.19	2.7	0.48	3.09^*^
0.2	0.58	4.47^**^	2.8	0.29	1.54	0.2	0.10	0.81	2.8	0.41	2.48
0.3	0.64	5.00^***^	2.9	0.22	1.20	0.3	0.17	1.54	2.9	0.42	2.35
0.4	0.60	4.30^**^	3.0	0.14	0.81	0.4	0.26	2.44	3.0	0.39	2.33
0.5	0.60	5.07^***^	3.1	0.06	0.36	0.5	0.24	2.40	3.1	0.35	2.19
0.6	0.50	3.81^**^	3.2	0.08	0.46	0.6	0.18	1.81	3.2	0.31	2.08
0.7	0.49	3.98^**^	3.3	0.27	1.91	0.7	0.10	1.06	3.3	0.41	2.87^*^
0.8	0.54	4.24^**^	3.4	0.27	1.90	0.8	0.14	1.42	3.4	0.49	3.11^*^
0.9	0.64	4.48^**^	3.5	0.25	1.68	0.9	0.12	1.19	3.5	0.50	3.05^*^
1.0	0.55	3.84^**^	3.6	0.19	1.32	1.0	0.03	0.30	3.6	0.48	2.82^*^
1.1	0.57	4.08^**^	3.7	0.16	1.09	1.1	−0.03	−0.23	3.7	0.51	3.00^*^
1.2	0.60	4.19^**^	3.8	0.08	0.55	1.2	−0.12	−0.98	3.8	0.39	2.60
1.3	0.63	4.17^**^	3.9	−0.02	−0.15	1.3	−0.11	−0.82	3.9	0.40	2.57
1.4	0.61	4.73^**^	4.0	0.00	0.03	1.4	−0.06	−0.58	4.0	0.41	2.59
1.5	0.57	4.50^**^	4.1	−0.04	−0.24	1.5	−0.03	−0.34	4.1	0.36	2.19
1.6	0.53	4.00^**^	4.2	−0.02	−0.18	1.6	0.01	0.14	4.2	0.30	1.94
1.7	0.58	4.41^**^	4.3	−0.02	−0.12	1.7	0.13	1.24	4.3	0.32	1.99
1.8	0.47	4.03^**^	4.4	−0.03	−0.25	1.8	0.06	0.56	4.4	0.44	2.89^*^
1.9	0.47	3.62^*^	4.5	0.06	0.54	1.9	0.03	0.25	4.5	0.42	2.60
2.0	0.53	4.07^**^	4.6	0.09	0.69	2.0	0.09	0.85	4.6	0.43	2.85^*^
2.1	0.43	2.96^*^	4.7	0.10	0.74	2.1	0.07	0.58	4.7	0.39	2.56
2.2	0.59	3.54^**^	4.8	0.08	0.64	2.2	0.15	1.02	4.8	0.33	2.19
2.3	0.49	2.72^*^	4.9	0.05	0.39	2.3	0.27	1.83	4.9	0.31	2.07
2.4	0.51	2.80^*^	5.0	0.03	0.25	2.4	0.34	2.19	5.0	0.29	1.86
2.5	0.53	3.12^**^				2.5	0.44	2.99^*^			

* p <.05;

** p <.01;

*** *p <.001*.

## Discussion

The goal of this study was to investigate whether multidimensional analysis of facial expression can be a suitable as basis for the in-vehicle measurement of the drivers' emotion. Especially, we were interested whether we can capture the dynamics of facial expressions by considering effects of appraisal components. We found that the facial expression indicators of high novelty and low power were significantly activated after fear events. Furthermore, after fear events, the activation of high novelty occurred earlier than the activation of low power.

According to the self-report the experimental manipulation was successful. The PANAS item “scared” had a higher value in Fear scenarios, while the participants' rating on the PANAS item “relax” was higher in BL. The results provided evidence that the induction of fear and relaxation was successful. The evidence for a successful manipulation of the experiment was also found in the SAM and novelty scales. Fear is supposed to be located lower on the power and the valence dimension and higher on the arousal and the novelty dimension (Fontaine et al., 2007; Gillioz et al., 2016). The subjective ratings on the dimension of valence were lower and the ratings on the dimension of arousal and novelty were higher in Fear than BL. Besides, the subjective ratings on the dimension of power were descriptively lower (not significantly, though). This may be due to the fact that the SAM's representativeness of emotional dimensions is still a question at issue (Schmidtke et al., 2014), so that the understanding of the SAM dimension of power could differ between participants. However, in total the manipulation check suggests that we successfully induced the emotional state of fear and relaxation in our driving simulator study.

According to the analysis of the time difference between AU compounds, we confirmed that facial expressions could be multidimensionally and dynamically analyzed. The activation of AU 1, 2, 4, and 5 was earlier than AU 20, 25, and 26. However, the activation of AU 7 and 15 was not as excepted. This may be due to the fact that the mapping between AUs and appraisal components is not always unique and AU7 and AU15 were also considered as the indicator of the appraisal component of unpleasantness (Scherer et al., 2018). Generally, it was revealed that the activation of the AUs in the upper face, which served as the indicator of high novelty, occurs earlier than the activation of the AUs in lower face, which served as the indicator of low power. On the one hand, the results on the dynamics of facial expression provided evidence for the existence of novelty and power appraisals as proposed by the CPM. On the other hand, the temporal difference between novelty and power appraisal was verified. It was consistent with the prediction of the CPM that the appraisal of novelty occurs earlier than the appraisal of power. The results of this study suggest that emotions could be multidimensionally dynamically assessed through different dimensions at different times.

Besides the multidimensional and dynamic interpretation of emotions, the CPM interprets the individual differences in emotional reactions. According to the CPM, emotions are triggered by individual appraisals, which depend on the individual's goals, values and coping potential (Scherer, 2009b). In other words, the same event could produce an emotion with different time course and intensity or even a different emotion. With regard to the difference in time, we used a 5 s time window to ensure that every onset of event-related facial expressions could be collected. The comparably small standard error for the components of the different experimental conditions suggests that the variance of the underlying individual appraisals was low in our study, which may be explained by the fact that the cover story and instructions ensured that participants had similar goals during the drives.

With respect to the ecological validity of this study, there are a few issues worth mentioning. We created an event producing a relatively strong emotional reaction in order use this strong reaction to evaluate whether it is in general possible to use the CPM to model facial reactions of drivers/users in a realistic setting (such as a driving simulation). We see this as a first step to employ the CPM as basis for in-vehicle emotion recognition and acknowledge that further research with less intense emotional episodes as well as during real-world driving is needed. In addition, a driving simulator setup with less ecological validity compared to real-world driving was chosen to have more control about the environmental conditions (e.g., weather). However, in general results from driving simulators have been shown to be transferable to real driving (see Shechtman et al., 2009; Helland et al., 2013). To add, the setting with the automated driving is a realistic setting given the current developments in the automotive domain, so that humans in vehicles will soon be able to engage in other tasks than controlling the car (like in the scenarios chosen).

In addition, it has to be noted that interpreting facial expressions alone is mostly not sufficient to know why the driver experiences a certain emotion and therefore also not sufficient to select the appropriate intervention strategy to support the driver. In a complex setting such as driving, we cannot say based on the facial expression alone whether the driver is fearful due to information she or he has received from a telephone conversation partner or due to the “risky” driving style of the automation. Therefore, it is also necessary to create a representation of the context to derive the need of the driver in a very situation as basis for the provision of the best possible intervention strategy (e.g., Drewitz et al., 2020). For instance, if appraisals pointing to fear have been detected, a virtual in-vehicle assistant could check whether parameters in the environment assessment, such as time-to-collision to the vehicle in front, indicate the occurrence of critical traffic events and, based on previous situations, could determine how likely the fear results from these. In case these probabilities are high, a specific intervention like a more defensive driving style could be chosen. If no relation to the vehicle exterior is likely and no other information about potential causes for the fearful state of the driver are present, the AI assistance could offer more general help or even ask for the cause. To sum up, a specific support of the user needs more information than solely the interpretation of the facial expressions, however, a detection of the facial expression is an important step to be able to interpret the emotions of the driver in the first place.

The main limitation of this study is the way fear was induced in the context of driving. A dynamic assessment of emotions requires an event-related analysis with a distinct onset. Thus, we needed to set up an emotional event to induce fear and define a time point as the onset of this event. In this study, the traffic accident was regarded as the event of fear and the SMS 5 s before the accident was regarded as a distraction, which was assumed to intensify the fear against the accident. However, according to the results, in which the AU compound of high novelty was already activated at the 0 s after the event onset (see Table 4), the appraisal component of high novelty might have started earlier than the accident itself due to the SMS. Hence, future work using this approach should consider an event with a more distinct onset. Additionally, although the CPM model would predict similar facial expressions when similar appraisals are experienced, this generalizability across different events, e.g., the facial expression after a traffic accident as used here compared to expressions after other fearful (with less intensity) or other emotional events (such as something surprising) with similar underlying novelty and power appraisals needs to be evaluated in future work.

Another limitation is reliability of the software used for coding of AU activations. In order to simulate the in-vehicle facial expression recognition at the application level, we used the software package FACET to automatically quantify changes of AUs. Although the software was confirmed to have a high positive correlation with EMG recordings (Kulke et al., 2020), it is assumed that the recognition performance of spontaneous facial expressions in video is much lower than in photos (Stöckli et al., 2018). Hence, in order to control the reliability of software coding, human coding could verify the performance of automated facial expression coding in future research.

## Conclusions

This research provides a new perspective on affective computing. For automated assessment, emotions were previously mostly regarded as a state with a single constant facial expression. However, facial expressions, especially in wild contexts such as driving, are dynamic processes resulting from underlying different appraisals. Models for emotion measurements from facial expressions need consider this multidimensional and dynamic nature. For the affective computing not only, the intensity and the duration of facial expressions is relevant, but also the temporal course of the activations of the different AUs in the facial expression, especially because only a minority of AUs can be unambiguously associated to specific emotions (Mehu and Scherer, 2015). Instead of chasing a certain pattern of facial expressions for a specific emotion, a dynamic perspective provides a multidimensional and multi-time domain solution, which can improve a robust and reliable measurement of drivers' emotion.

## Data Availability Statement

The dataset analyzed during the current study is available from the corresponding author on reasonable request.

## Ethics Statement

Ethical review and approval was not required for the study on human participants in accordance with the local legislation and institutional requirements. The patients/participants provided their written informed consent to participate in this study.

## Author Contributions

MZ, KI, and UD: conceptualization and methodology. MZ: data curation, investigation, visualization, and writing – original draft. KI: project administration. KI, UD, and MJ: supervision. MZ, KI, UD, and MJ: writing – review & editing. All authors contributed to the article and approved the submitted version.

## Conflict of Interest

The authors declare that the research was conducted in the absence of any commercial or financial relationships that could be construed as a potential conflict of interest.
